# Exploring differences in perceptions of gentrification, neighborhood satisfaction, social cohesion, and health among residents of two predominantly African American Pittsburgh neighborhoods (n = 60)

**DOI:** 10.1186/s12889-023-16970-4

**Published:** 2023-11-01

**Authors:** Alexandra Mendoza-Graf, Sarah MacCarthy, Rebecca Collins, La’Vette Wagner, Tamara Dubowitz

**Affiliations:** 1 RAND Corporation, 1776 Main Street, Santa Monica, CA 90407 USA; 2 Department of Health Behavior, University of Alabama School of Public Health, 227 Ryals Public Health Building, 1665 University Boulevard, Birmingham, Alabama 35233 England; 3 RAND Corporation, Pittsburgh Office, 1776 Main Street, Santa Monica, CA 90407 USA

**Keywords:** Gentrification, Social cohesion, Neighborhood satisfaction, Health, Qualitative

## Abstract

**Background:**

Gentrification often leads to changes in the social and physical environment of neighborhoods, which social capital theory has found are connected to aspects of resident health and wellbeing. A growing body of literature has explored the impact of gentrification on health and wellbeing of residents. The goal of this study is to qualitatively explore the ways in which gentrification may have impacted perceptions of neighborhood satisfaction, social cohesion, and health of neighborhood residents (n = 60) from two predominantly Black neighborhoods in Pittsburgh, Pennsylvania, one of which experienced Black gentrification during the study’s time period. This analysis is unique in its ability to capture experiences of residents who remained in their neighborhood throughout the course of the study, as well as those who moved away from their neighborhood.

**Methods:**

Participants were randomly selected from a larger cohort enrolled in a quasi-experimental study and categorized by whether they lived in a census tract that gentrified, whether they owned or rented their home, and whether they moved from the neighborhood or remained in the same place of residence between 2011 and 2018. Phone interviews lasting approximately 30 min were conducted with participants and were audio recorded and transcribed verbatim. Participants were provided a $40 gift card for their time. Interview data were analyzed using a directed content approach, and Cohen’s Kappa was obtained (k = 0.924) to signal good inter-rater reliability.

**Results:**

Results showed renters in gentrified census tracts overwhelmingly viewed gentrification trends as a negative change compared to homeowners. Overall, participants from gentrified census tracts reported being relatively satisfied with their neighborhood, though some suggested there were fewer resources in the neighborhood over time; felt their social cohesion had deteriorated over time; and more commonly reflected negative health changes over time.

**Conclusions:**

These findings suggest that while gentrification can bring much needed improvements to neighborhoods, it can also bring other disruptive changes that affect the health and wellbeing of existing residents.

**Supplementary Information:**

The online version contains supplementary material available at 10.1186/s12889-023-16970-4.

## Background

Gentrification represents an urban process that has the potential to change basic social and physical structures within neighborhoods (e.g., increasing home values, neighborhood demographic changes, revitalization of housing and other neighborhood structures). This is important because research has shown that health and wellbeing can be impacted by the social and physical aspects of one’s neighborhood environment [1–3]. Factors that can typically change in gentrifying settings include: access to food, greenspace, and affordable housing; social support, cohesion, and networks; and feelings of safety, prejudice, or discrimination [4]. Social capital theory [5, 6] suggests that cumulative and transient exposure to factors such as safety, resources (e.g., libraries, recreational facilities, grocery stores, and social services), and social connections are mechanisms through which a change in the neighborhood environment can impact health and wellbeing [4, 7]. Specifically, these factors have been found to affect health through their impact on healthcare utilization, health related behaviors, and biological responses [4]. Given the connections described in social capital theory, there is potential for gentrification to impact changes in health and wellbeing.

Though the definition of gentrification and how to measure it is often disputed, for the purposes of this study, we define it as the process through which disinvested neighborhoods experience renewal, driven by an influx in college educated individuals and upwardly trending housing prices [8–10]. Further, there continues to be disagreement about whether neighborhood changes brought about by gentrification are generally positive or negative for the health and wellbeing of its residents. Some potentially beneficial changes stemming from gentrification may include increases in investments and redevelopment into disinvested urban neighborhoods, making the neighborhoods more attractive to middle-income households [11]. Additionally, gentrification may bring about improvements in the residential environment for those who remain in their neighborhood, compared to those from low socioeconomic status neighborhoods that do not gentrify [12]. On the other hand, studies have warned that gentrification can lead to displacement of residents [13–18] and increased outmigration for less educated renters [19], resulting in a higher likelihood of economically disadvantaged residents moving to lower-income neighborhoods [20].

Given the potential for gentrification to change the social and physical aspects of neighborhood environments, a growing body of literature seeks to explore various health [21–36] and wellbeing [12, 18, 26, 37–48] factors that may be impacted for residents living in gentrifying neighborhoods. Specifically, qualitative research in this space has described a number of health and wellbeing outcomes that have been found within the context of gentrification, including diminished food security for some residents in gentrifying areas [28, 49]; the loss of social support [50], social ties [41, 51], and cohesion [41] for some existing residents; as well as cultural displacement [18] and racial discrimination [52]. Other quantitative work in this area has shown mixed findings for other aspects of health, like mental health, where some studies have found no associations between gentrification and psychotic episodes [33] nor in changes in psychological distress [53]; some have found poorer mental health for higher-income older adults in gentrifying neighborhoods compared to similar adults in low-income neighborhoods [22]; and yet others have found higher degrees of psychological distress for residents in low-income neighborhoods when compared to gentrifying neighborhoods [31]. These findings should be considered with the caveat that research on gentrification is inherently difficult to conduct, given the difficulty associated with tracking neighborhood residents over time, particularly those who have moved or been displaced.

This research capitalizes on data from a cohort of residents from two neighborhoods in Pittsburgh, Pennsylvania, that are part of a quasi-experimental study, one of which went through processes of gentrification during the study period. This parent quasi-experimental study is unique in that from its original cohort, it continued to follow participants who moved out of the neighborhood, which has not been widely captured in research related to gentrification and health to date. Prior multivariate analyses on the parent study sample suggested differential changes in neighborhood satisfaction and social cohesion over time by whether participants lived in a census tract that gentrified or not, as well as differences in neighborhood satisfaction changes by whether participants owned or rented their homes and whether they moved or remained in the same place of residence during the study period. The current study qualitatively explores the ways in which various changes during the gentrification process may have impacted the health and wellbeing of a subset of neighborhood residents, when compared to those from the other study neighborhood that did not experience gentrification. It uses semi-structured interviews with a subset of participants who were part of the parent study between 2011 and 2018 to understand their opinions of gentrification, neighborhood satisfaction, social cohesion, and health, with attention to differences by gentrification, homeownership, and mover status. This work adds to the current qualitative literature in this space by exploring various aspects of health and wellbeing that have been documented to be impacted by gentrification in other settings, and it also draws on and provides context for prior empirical results [8] as well as conceptual frameworks outlining how changes in the social and physical neighborhood environment brought about gentrification are connected to changes in health outcomes [4].

## Methods

### Parent study details

The Pittsburgh Hill/Homewood Research on Neighborhood Change and Health (PHRESH) study is a longitudinal, quasi-experimental study located in two neighborhoods in Pittsburgh, Hill District and Homewood (R01CA149105) which began in 2011. The study was designed to understand how changes in the social and physical environment impact health outcomes and other factors among neighborhood residents. At the beginning of the study, both of the neighborhoods were low-income and predominantly Black. During the course of the study, the Hill District experienced substantial increases in neighborhood investments, through the establishment of a full-service supermarket (the first one since the 1980s), the re-development of public housing (including the replacement of some public housing units with mixed-income housing), the renovation and creation of neighborhood greenspace (including several new parks and trails connecting parks), and other commercial investments (including the redevelopment of “Main Street”) [54–57]. The parent study participants were selected through a random sample in both neighborhoods, where data collectors enrolled participants through door-to-door recruitment [54]. The total study sample at 2011 was 1,372 and 597 of those same households were interviewed again in 2018.

There are a total of 13 census tracts between the two study neighborhoods. In the case of the two study neighborhoods, the census tract boundaries align with the boundaries of the neighborhoods as designated by the city. Across those 13 census tracts, 5 experienced gentrification in the form of Black gentrification between 2011 and 2016. Black gentrification specifically describes a gentrification process in which the influx of higher educated residents to a neighborhood is characterized by an increase in Black college educated individuals (rather than the more commonly considered change of increased White college educated individuals moving into a neighborhood) [21]. All census tracts that experienced gentrification were part of the Hill District neighborhood, the neighborhood that received substantial increases in investments over the study period [53].

### Sampling and data collection procedures

**Fig. 1:**
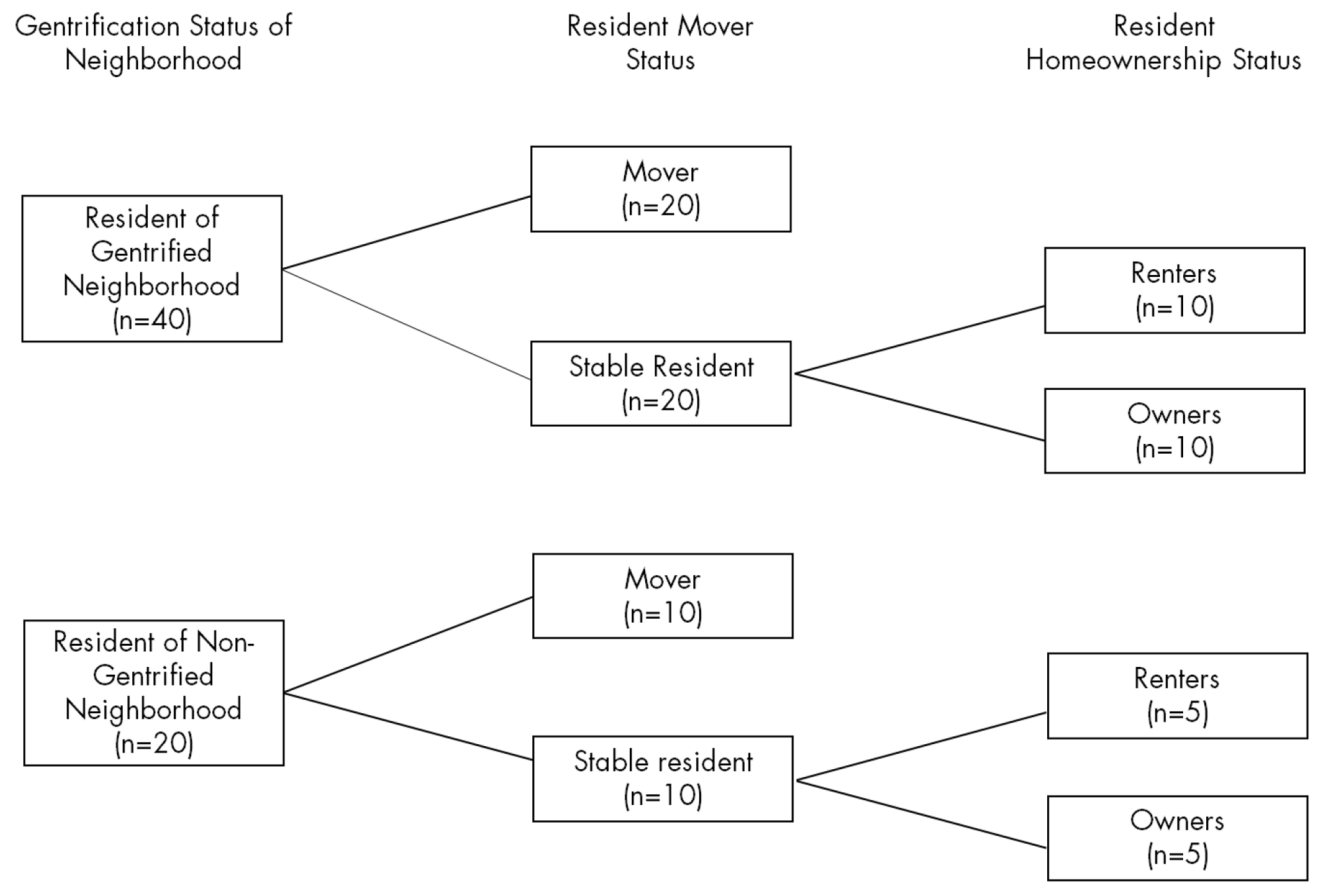
Qualitative interview participant breakdown

For this study participants in 2018 (i.e., those who responded to an interviewer-administered survey) were selected into categories stratified by: gentrification status (i.e., whether participants lived in study census tract, henceforth referred to as tract, that gentrified between 2011 and 2018), which was determined using a modified version of a measure developed by Freeman [9, 10], with a more detailed description of this measure found in our prior analyses [53]; homeownership status (i.e., whether a participant owned or rented their home between 2011 and 2018), and moving status (i.e., whether participants moved or remained in the same place of residence between 2011 and 2018, hereafter referred to as movers and stable residents) (Fig. 1). From these categories, we then randomly selected participants within each group for interview recruitment.

Recruitment for the interviews was conducted by the cisgender female, African American Field Coordinator of the parent study, who was raised in one of the study neighborhoods (and also had extensive engagement with parent study participants during the entirety of the study [2011–2021]). Participants were contacted by phone and asked to schedule interviews. Of the 180 participants who were contacted, 4 were deceased, 5 refused (either at the time of recruitment or at the time of consent), 108 could not be reached (83 did not answer their phone, 25 had a number listed that was out of service), and 63 participants agreed to do an interview (60 participated in the interview, and 3 were no-shows for their scheduled interview times). We recruited 40 participants from gentrified tracts and 20 participants from tracts that did not experience gentrification during the study period. Based on our prior analyses, we saw relatively more variation in terms of neighborhood change occurring in the gentrifying tracts and felt we needed to interview more participants from gentrified tracts to reach data saturation, which is why we interviewed larger pool of participants from the gentrified tracts. In general, the study population is one that has been difficult to reach in prior data collection efforts. This is likely due to the fact that the larger parent study sample is predominantly older, low-income, and study participants often face issues with lack of telephone service, frequent illness, multiple jobs, or other competing responsibilities. However, we compared descriptive demographic information of those who participated in the interviews and those who could not be reached, and there were no statistically significant differences between the two groups. The final breakdown of interviews can be seen in Fig. 1, broken down by our categories of interest. The interviewer (AMG) was a cisgender female Latina researcher with experience and training in qualitative data collection and analysis with community members. A total of 60 phone interviews were conducted with PHRESH study participants between February and March of 2021. Participants provided verbal consent, as was approved by the RAND Corporation’s IRB, and interviews were digitally recorded and transcribed verbatim. The duration of interviews was between 20 and 45 min, with most close to 30 min in length. Each participant was mailed a $40 grocery store gift card as compensation for their time. A description of participants’ demographic characteristics can be viewed in Table 1.

**Table 1:** Interview Participants’ Demographic Characteristics

Characteristics	All Participants (n = 60)	Gentrified Tracts (n = 40)	Non-gentrified Tracts (n = 20)	Movers (n = 30)	Stable Residents (n = 30)	Renters (n = 41)	Owners (n = 19)
**Age** (mean)	62.1	62.1	62.2	58.0	66.2	61.0	66.3
**Female**	87%	88%	85%	87%	87%	90%	78%
**Employment**							
*Full time*	32%	30%	35%	20%	43%	28%	39%
*Part time*	12%	13%	10%	10%	13%	10%	17%
*Unemployed*	7%	8%	5%	13%	0%	10%	0%
*Retired*	17%	20%	10%	13%	20%	18%	17%
*Disabled*	22%	18%	30%	33%	10%	25%	11%
*Other*	12%	13%	10%	10%	13%	10%	17%
**Any kids in the household**	27%	28%	25%	30%	23%	33%	17%
**Married or living with a partner**	23%	23%	25%	13%	33%	20%	33%
Per capita household Income**							
*Less than $5,000*	27%	23%	35%	37%	17%	38%	0%
*$5,000–9,999*	22%	15%	35%	23%	20%	28%	11%
*$10,000–19,999*	27%	35%	10%	20%	33%	20%	39%
*$ 20,000 or more*	25%	28%	20%	20%	30%	15%	50%
Educational Attainment*							
*Less than High School*	17%	20%	10%	30%	3%	25%	0%
*High School*	42%	38%	50%	30%	53%	35%	56%
*Some College/Tech*	32%	35%	25%	30%	33%	30%	33%
*College/Graduate Degree*	10%	8%	15%	10%	10%	10%	11%

Demographic characteristics in are from Wave 2 of the study survey (2013), since some participants did not enter the study until Wave 2. Age was adjusted to represent mean age at 2021. **The difference for household income by home ownership was statistically significant at p < 0.001.*The difference for educational attainment by home ownership was statistically significant at p < 0.05

The interview protocol contained questions about gentrification, satisfaction with their neighborhood, and changes in social cohesion and health, as well as the social context in which these processes occurred. To understand participant views on gentrification, they were read a description of gentrification trends (e.g., increases in housing costs, new businesses coming into the neighborhood, and more people moving out/new, higher-income people moving into the area) and asked whether they felt those trends were happening in their neighborhood. They were also asked about their opinions of such trends and whether they were familiar with the term *gentrification.* To understand participants’ satisfaction with their neighborhood, they were asked their thoughts about the neighborhood trajectory; the most and least favorite aspects of their neighborhood; and opinions about changes in the neighborhood over time. To understand any changes in social cohesion brought about by changes in participants’ neighborhoods, they were asked whether they had changes in the people they know or their close relationships within their neighborhood and whether and how they like to engage in their communities. Further, for understanding whether there were any health changes that participants associated with changes in their neighborhood, they were first recounted changes they had described in the neighborhood and asked whether they felt these changes had impacted any aspect of their health, including their diet/how they get their groceries, their stress, how they get care, or any other aspect of health they could think of. Finally, interviews with participants were conducted during the COVID-19 pandemic and in the aftermath of several protest related to the Black Lives Matter (BLM) movement. These factors were thought to potentially also impact participants’ perceptions of their neighborhood (since there may have been protests related to BLM in their neighborhoods or potential closures of local resources due to the COVID-19 pandemic); changes social cohesion (since the COVID-19 pandemic may have led to people keeping more to themselves); and changes in health (due to the risks and implications of contracting the COVID-19 virus). Due to these contextual factors occurring around the time of data collection, participants were also asked about whether they had noticed any changes in their neighborhood or in their personal lives due to these two factors.

### Analysis

The interview data were analyzed using a directed content approach [58]. Transcripts were reviewed by two researchers with extensive qualitative expertise (AMG and SM) to identify initial themes. An inductive and deductive approach was taken for developing an initial codebook, using both the interview protocol and initial themes to inform the components in the codebook. The codebook was shared with the rest of the research team (TD, BC, LW) to obtain input on the salience of preliminary themes. The two researchers then jointly coded 12 transcripts using Dedoose qualitative software [59] and made modifications and additions to the codebook as needed. The final codebook contained 4 codes and 17 sub-codes, as well as definitions, inclusion criteria, and example text to assist with coding determinations. Interrater reliability was established using Cohen’s Kappa on a set of 52 excerpts. Cohen’s Kappa was 0.924 representing good interrater reliability. The remaining transcripts were individually coded, and AMG and SM met weekly to discuss any concerns that came up during the coding process. Upon completion of the coding, excerpts for each code were exported to Excel and reviewed to understand the range and frequency of themes, identify additional themes that emerged during the coding process, and to understand any differences by participant type (e.g., gentrification status, homeownership status, and mover status).

## Results

The findings from the semi-structured interviews are organized according to the various factors thought to be related to gentrification explored in our prior analyses of the full study sample. This included participants providing their perspectives on gentrification trends in their neighborhoods, satisfaction with their neighborhood, and changes in social cohesion and health, as well as the social context in which these processes occurred. Differences among participants were found on some of these topics based on gentrification status, on whether they moved to another home during the study period, or whether they rented or owned their homes. Sub-group differences on these topics are noted throughout the results, and there were otherwise no notable differences found between participants among the different categories of interest (e.g., gentrification status, mover status, and homeownership status). Exemplary quotes were included through the text to illustrate some of the most salient themes that came up during the interviews and additional quotes can be found in Supplementary Tables 1, 2, 3, and 4. Additionally, a total of 7 participants (6 from gentrified tracts and 1 from a non-gentrified tract) who moved during the study period were forced to move due to housing renovations, and we note cases where there were any themes that were specific to this population.

### Gentrification

Conversations about gentrification revealed little understanding of the term among participants, divisions between those in gentrified and non-gentrified tracts on perceptions of change in their neighborhoods, and disagreements between renters and owners on whether these types of trends were generally positive or negative.

Unexpectedly, when participants were asked about the term gentrification, almost none were familiar with the term. Once described, however, the majority of respondents from gentrifying tracts felt gentrification trends (i.e., increased cost of living, new businesses coming to the neighborhood, people moving out and wealthier people moving in) were happening in their neighborhood and agreed that there had been increases in the cost of living and in residential turnover. There was also a recognition that these types of changes placed an additional burden on people of color who had been living in the neighborhood for a number of years, with some specifying that the burden was higher for those who were lower-income.

*You know, the home[s] is so* expensive, *a lot of times people of color can’t afford it…the Hill used to be a mixture. It’s a mixture now. But like I said, the homes, the people that bought these houses, at first, they’re not even there anymore. So, this is just different.*

*Participant from gentrified tract, renter, stable resident*

*They want to come to a place where they can get closer, get to work quicker instead of driving all the way somewhere. So, they’re trying to force the blacks out. Not to be racist or anything, but they are, and they can get more money from one of these college kids, somebody who’s working downtown, somebody who’s making some good money. They don’t mind paying the $1,400 rent. So, it’s like they don’t really want too many black folks up here unless you really got some money really. But otherwise, they’re trying to force us out.*

*Participant from gentrified tract, renter, mover*

Further, participants from gentrified tracts were somewhat evenly divided on how they felt about such changes by homeownership status, where renters viewed gentrification as trend that was detrimental to the neighborhood, explaining that the types of changes (i.e., higher costs of living and increased residential turnover) did not seem fair and that the higher prices pushed longtime residents out, and homeowners mostly felt gentrification was a positive change for the neighborhood and relayed the changes may bring more jobs to the area, help to keep up the neighborhood, attract more people and diversity to the neighborhood, and potentially bring more businesses to the area. One renter shared their negative sentiments about gentrification trend, while a homeowner described the benefits of such trends:

*I’ve been in the Hill all my life. So, stop chasing people who’ve been here all their lives out. You know, you build a new house and the person who’s been living in the Hill can’t afford it anymore. Where are they supposed to go? They are pushing you out in the suburbs. That’s not where I’m from. And that’s not where I want to be.*

*Participant from gentrified tract, renter, stable resident*

*I think that that’s a good thing because, in order to interest people to come into the area, it has to look decent. And nobody wants to come in and live in beat-down stuff. So, I think that that’s really good. I think that for your neighborhood to look nice you have to keep improving it.*

*Participant from gentrified tract, owner, stable resident*

There was less alignment among participants in non-gentrifying tracts, but of those who felt gentrification was happening, most attributed it the construction of new housing, and said they either would not mind or felt positively about the idea of gentrification happening in their neighborhood. Despite the differences in opinions about gentrification, almost all expressed they would want to remain in their neighborhoods regardless of any kind of gentrification trends. Some in particular expressed the importance of being able to stay in their historically Black neighborhoods:

*Y’all take our children, y’all take our jobs, y’all take our men, y’all take our lives. Now y’all want to take our homes. What more do y’all want?…We built this foundation. Anything that’s over here, we built it, we loved it. Now, y’all want to come over here and take over for what? Come on now!*

*Participant from gentrified tract, renter, mover*

### Neighborhood satisfaction

Neighborhood satisfaction was explored through the lens of the perceived trajectory of the neighborhood, as well as residents’ most and least favorite aspects of their neighborhood. Differences were found between those from gentrified tracts and non-gentrified tracts on whether they felt their neighborhoods were on a positive trajectory. For most participants, regardless of gentrification, mover, or homeownership status, their favorite aspect of their neighborhood seemed to be the convenience to downtown and other resources, while their least favorite aspect included more variation, with some aspects being common across gentrification status and others differing by gentrification status.

*Trajectory of the neighborhood.* While responses from participants living in non-gentrified census tracts varied, participants from gentrified census tracts generally felt their neighborhood was on a positive trajectory. One nuance to these findings, however, was that a few participants from gentrified tracts felt this was only the case for certain groups of people (e.g., higher income, non-Black residents).

*They don’t treat low income the way they treat the higher pay, rich people. And it’s not supposed to be that. That’s discrimination. And it happens every day, but we as Black people are afraid of authority and will not speak up.*

*Participant from gentrified tract, renter, mover*

*Most favorite aspects of neighborhoods.* Across both types of tracts, participants reflected positively on their neighborhood’s convenience. Participants from gentrified neighborhoods often said they appreciated the safety and quiet, the housing improvements, the increased police presence, and to a lesser extent, the diversity and political progressiveness of the neighborhood. One participant who was particularly enthusiastic about improvements in housing shared:

*They did the greatest thing. They remodeled. They turned apartments into houses. That was good. So, we can keep our grandkids and things. We got houses now up there. I felt really good about it, excellent. That was long time overdue. We all need it.*

*Participant from gentrified tract, renter, mover*

Residents did share some caveats about the improvements to housing, including: some residents not being able to move back to their buildings; the closure of local businesses to clear the way for the construction; and diminished affordability and quality (one person even stating the walls were separating from one another in one room). Some felt that since some buildings were transformed into mixed-income housing, the new or renovated housing no longer catered to just low-income populations, leading some social services to leave the area. Further, some of the mixed-income housing seemed to separate residents into different sections and buildings, and residents felt the upkeep and quality of the low-income parts was subpar. Finally, it was shared that some of the rules and regulations of the new housing developments were too strict (e.g., people were not allowed to sit out on stoops, children could not ride bikes in the building areas, etc.). Only residents in non-gentrifying tracts mentioned appreciating the people and neighbors in the area, as they felt this contributed to having a good community.

*Least favorite aspects of neighborhoods.* Neighborhood aspects that were disliked from participants across gentrified and non-gentrified tracts included: violence in the neighborhood (e.g., shooting and fighting); the lack of resources and activities available for children and seniors (e.g., good playgrounds, places for recreation, places to sit down and eat, etc.); the departure of the grocery store in the Hill District (*note that during the study, the full-service supermarket opened in the neighborhood that went through gentrification in 2013, but shuttered in 2019*); and all of the vacant properties. The perception that there were fewer resources in participants’ neighborhood was particularly prevalent among permanent residents, but overall, most participants said they had to leave the neighborhood to access needed resources, and this was particularly pronounced for participants in gentrifying tracts once the grocery store closed. One participant from a gentrified tract who felt there were fewer resources in the neighborhood shared that:

*You ain’t got nowhere to eat over here and I think that’s terrible. That’s my thing, stuff like that. Not even for these kids, they had no recreation like we had, they ain’t got nothing over here for these kids nothing and then you all want to sit up and talk about it. They hanging out, well they ain’t got nothing to do…When we were all kids, we had three recreation centers on one street.*

*Participant from gentrified tract, renter, stable resident*

Participants from the gentrified tracts also said they disliked the lack of maintenance (e.g., of buildings, sidewalks, and streets); issues of drug use; and the perceived lack of responsiveness from local representatives. Participants from the non-gentrified tracts disliked the lack of police presence, and issues with people coming into the neighborhood from other areas to cause trouble.

### Social cohesion changes

Social cohesion was explored through the lens of neighborhood interactions, as well as community engagement. In terms of neighborhood interactions, most participants felt they had some close relationships in the neighborhood, but there was variation by gentrification status on the frequency and quality of interactions with people in their neighborhood over time. Further, differences were found between renters and homeowners on whether they liked to engage in their neighborhoods, and this also sometimes varied by gentrification status.

*Neighborhood Interactions.* Overall, the majority of participants in both types of tracts said they had at least some close relationships or people they could turn to in the neighborhood if they needed help. Contradictingly, however, there were also comments from participants in both types of tracts saying new residents moving into the neighborhood did not seem to be as friendly or willing to come together as prior residents. In gentrified tracts, some shared they mostly kept to themselves and did not know many people despite having lived in the area for years, while others felt they knew almost everyone in their neighborhood. Similarly, in non-gentrified tracts, some participants said they knew and interacted with a lot of people and others said they prefer to keep to themselves. With regard to feeling like newer neighbors were less friendly, several participants from gentrified tracts recounted:

*It used to be, people in the neighborhood looked out for each other, you know?… I was telling you like my nephew passed away…he was just getting out of his car and just passed out. On the street, nobody came around…They stood across the street where I live, just looking…But they didn’t say anything.*

*Participant from gentrified tract, owner, stable resident*

*The only time you see somebody is if something happens, they might look out the door. But you don’t see people unless there’s something bad. People don’t bother… I’m used to, “How you doing? Good morning.” Whatever. And people just don’t do it, they just don’t care.*

*Participant from gentrified tract, owner, stable resident*

*I still have close friends, but I just I mean I’m not close with a whole lot of people. When I was growing up, everybody knew everybody and we all got along, but now that I’m older like I said some moved away, some are dead, some we kind of drifted apart as our lives changed…Back then, people looked out for one another. You don’t have that anymore.*

*Participant from gentrified tract, owner, stable resident*

In both types of tracts, several participants who moved away suggested they were not very close with people in their new neighborhoods. Additionally, some residents who moved as a result of being forced to move when there was renovation of housing in their neighborhood shared the difficulties of living in a place that was not their first choice. One older resident who moved due to housing renovations described the difficulties it caused:

*Up there, it’s isolated up there. It’s up on top of the hill. You have to leave from up there to get any type of services, anything. You know, I hated every second of that project up there, yeah… I didn’t – I just didn’t associate too much with the people. I worked up there on the polls in Arlington, and that was about it. You know, they have nothing for the seniors up there. Most of the stuff was geared towards the young kids, you know.*

*Participant from gentrified tract, renter, mover*

Some differences emerged by gentrification status, where participants from gentrified tracts mostly said they had fewer interactions with people in their neighborhood over time due to fewer neighborhood activities; having to move away themselves; others moving away and new people moving in; houses in the neighborhood being torn down; or more strict restrictions in newer housing complexes (e.g., not being able to all sit out and watch kids play or ride their bikes because they have to play outside of the property). One participant who felt there were fewer neighborhood activities shared the difficulty it brought for socializing:

*I think that there’s new housing, but there’s not a lot of activities or things to do in a neighborhood. So, I think that people moving into the neighborhood are still doing things, wherever they came from because there’s nothing to do here. So, you’re really not meeting them and socializing with them…It was different before.*

*Participant from gentrified neighborhood, owner, stable resident*

*Community Engagement.* The majority of participants across both types of tracts said they like to engage in their respective communities, though this was somewhat more commonly heard from participants in the non-gentrified tracts. Within the respective types of tracts, one nuance was that there were differences in opinions about this between renters and homeowners, where a much higher proportion of homeowners said they liked to engage in their community than renters. Some homeowners in gentrified tracts, however, felt it had become more difficult to engage in their communities because information about neighborhood changes was not always readily available nor were residents always included in conversations or decisions about changes in the neighborhood.

*I think that there are changes happening. But I think that the meetings are held at strange times, so everybody can’t participate in them. And I think that it’s a handful of people who are making decisions or suggesting things, but it’s not open to the community. It’s very hard to find out about things. And, when you do find out, everything’s already in gear.*

*Participant from gentrified neighborhood, owner, stable resident*

Further, several participants from non-gentrified tracts felt there was lack of community togetherness and community meetings.

*It’s about the whole community coming together and stick together to make a change, we make a change when you come together, that’s when things change, but when you separate, then you can’t resolve anything. So that’s the main problem, people don’t want to come together, they all complain, but they don’t want to come together.*

*Participant from non-gentrified neighborhood, renter, stable resident*

### Health changes

Participants had a range of responses they provided with respect to changes in their health. More often participants from gentrified tracts indicated that their health had been impacted negatively due to neighborhood changes and commonly described diminished ability to exercise due to changes in the neighborhood, as well as an increase in stress, often due to having to move to another home. One participant who had to relocate during a renovation shared the difficult experience of having to move somewhere that was unsafe:

*Living in the projects can make you or break you…And it plays a little on your psyche…when I found out I was able to move back [after renovations], I was never so happy. It seemed like the whole time that I lived up in [the projects], there was a shooting every day, every day, there was some type of violence. I remember on Christmas Eve; they had a whole shootout in my court…they shot up the steps and the concrete steps fell. Like it was so much crazy. A guy I went to school with, I watched him die outside…And I saved someone from dying. Like it was a lot.*

*Participant from gentrified neighborhood, renter, mover*

Other commonly mentioned reasons for health changes were related to changes in neighborhood resources over the last several years, including difficulties with getting groceries, accessing a pharmacy, and accessing other social services, due to the recent closure of the only grocery store in the neighborhood and the departure of services catering to lower-income populations. On the other hand, participants from non-gentrified tracts mostly felt their health did not change much over time.

### Context of COVID-19 and the black lives matter movement

Important contextual events occurred during the course of this study (February through March of 2021). The COVID-19 pandemic prompted participants to reflect on changes in access to resources and sense of community connection. Additionally, many noted that COVID-19 impacted either their own health or that of their family. A few people shared feeling very isolated and depressed over not being able to spend time with friends, family or neighbors or not being able to see spouses or other family living in nursing homes. Others relayed feeling more anxious due to having lost their jobs during the pandemic. Some participants also mentioned changes in their physical activity either due to the closure of fitness centers or to hesitancy around being out in public. The BLM movement, which came to somewhat of a tipping point with the killing of George Floyd in May of 2020, generated less of a response among participants, though some felt that more attention was now being paid to issues related to the BLM movement. Some also felt there was more police presence due to the BLM movement, though there were disagreements about whether this was favorable for the community, and there were also others who expressed a displeasure with the looting that occurred during some marches, as it reminded them of looting that negatively impacted their communities after the death of Dr. Martin Luther King Jr.

## Discussion

The goal of this study was to better understand differences, including the processes and mechanisms, found in prior analyses on the relationship between gentrification and neighborhood satisfaction, social cohesion, and health among study participants from two neighborhoods, one of which experienced gentrification [8]. Results from the interviews suggested some differences among participants by gentrification, homeownership, and moving status.

Renters in gentrifying tracts overwhelmingly viewed gentrification trends in the neighborhood as a negative change, while homeowners were more likely to view gentrification trends positively. This is consistent with other studies finding homeowners being more likely to approve of changes in gentrifying neighborhoods than renters [60, 61]. These differences make sense if we consider that gentrification trends are often accompanied by rising housing prices, which for homeowners translates into increased home values, while for renters, this will often manifest in rising rents, creating added financial pressures for renters.

Overall, participants from gentrified census tracts felt their neighborhood was generally on a positive trajectory compared to those from tracts that did not gentrify. Despite their feelings about general neighborhood trajectories, permanent residents from gentrified tracts more commonly suggested they had fewer resources in their neighborhood than before, when compared to those who moved to another place of residence. These findings seem to align with other studies that showed increases in satisfaction in gentrifying neighborhoods compared to low-income neighborhoods [42] and lower satisfaction for long-term residents of gentrifying neighborhoods versus newer residents [43]. These seemingly conflicting findings could be explained by the fact that although gentrification can bring about needed improvements to an area, it can also impact the resource landscape of a neighborhood, changing the availability and accessibility of resources for more permanent residents.

Participants from gentrified tracts mostly felt they had fewer interactions with neighbors, were not as close to people in the neighborhood, and that newer, younger residents were not as friendly as others in the past. These findings align with others showing that gentrification resulted in residents experiencing disruptions in social ties and lack of inter-generational cohesion [41], as well as smaller increases in social cohesion [53]. This may be due to the fact that changes happening in gentrifying neighborhoods, such as residential turnover, can break social ties for existing residents but also make it difficult to build new relationships with incoming residents when turnover occurs more frequently.

Participants referenced changes in mental health as well as other aspects of health, especially due to moving. Further, participants from gentrified tracts more commonly reflected negative health changes because of decreases in neighborhood resources over time and closures related to the COVID-19 pandemic, whereas health changes in non-gentrified tracts were more commonly due to issues of safety and violence in the community. These findings are somewhat different from others in the literature showing gentrification is associated with improved aspects of health, such as self-rated health [21–24, 31] and hypertension [29]. Rather, our findings seem to align with studies that find more detrimental health outcomes for Black residents in gentrifying neighborhoods [21], and particularly with regard to self-rated health [31]. These findings should be considered within the context of the historical discrimination of Black populations with regard to land use, housing, and planning policy, which to this day contribute to differential outcomes for Black populations.

Taken together, these findings paint a complex picture of the experience of gentrification for Black populations in this specific Pittsburgh neighborhood. Overall, it seems that participants who were renters from gentrified census tracts were more likely to feel the pressures of rising housing costs and generally felt less engaged in community activities and decisions, and they were also more likely to have moved to another residence during the study period, which contributed to a sense of disconnect from their community. These experiences differed from participants who were homeowners from gentrified tracts, in that the homeowners were less likely to feel pressures from rising housing costs and therefore seemed to have more of an opportunity to embrace some gentrification changes and benefit from neighborhood improvements. Both renters and homeowners in gentrified tracts, however, seemed to be negatively impacted from resources leaving the neighborhood over time, suggesting there may be some aspects of gentrification that disproportionately affect sub-segments of a neighborhood, while other aspects can have a broader impact on most neighborhood residents.

### Limitations and strengths

There was a high number of non-respondents during recruitment, making it possible that participants were somehow different from those who did not respond. It is also possible that the interview results were impacted by other external events happening at the time besides gentrification, including the COVID-19 pandemic and the BLM Movement. Additionally, given the qualitative nature of the study and the fact that this research explored potential impacts of gentrification, and particularly Black gentrification, in a predominantly Black neighborhood, the results may not be applicable to other settings beyond the parent study population or other similar types of neighborhoods, and it cannot establish causal relationships between gentrification and neighborhood satisfaction, social cohesion, and health. Another aspect that could impact the applicability of these findings to other settings is the fact that there were a large number of planned investments made in the Hill District, which may make the gentrification experience different than other neighborhoods where gentrification is more so driven by migration patterns and market forces. Further, although this is a study about the relationship between gentrification and the health and wellbeing of residents, residents were largely not familiar with the term “gentrification” (though they did think there were significant changes happening in the neighborhood). It is possible that categorizing areas as “gentrified” may sometimes be a term imposed on a community, and greater efforts should be made to understand how community residents characterize the changes happening in their neighborhoods. Finally, given that we knew prior to the interviews that there was not much of a change in the racial makeup of the neighborhood, we did not specifically probe on racial aspects of gentrification typically considered, such as racialized housing, though some perceptions about how neighborhood changes related to race were brought up in participants’ responses. Future studies should consider probing on differential experiences of gentrification related to race even for instances of gentrification that do not feature much change in the racial makeup of the neighborhood. This study, however, provides a unique perspective on the potential impacts of Black gentrification in predominantly Black neighborhoods, particularly due to the ability to obtain perspectives from both residents who remained in gentrified census tracts as well as those who moved away during the process of gentrification.

## Conclusion

Findings from this study provide context to help further understand what aspects of health and wellbeing may be more impacted by neighborhood change for Black residents and showcase potential areas for future research. Given our findings, there are a few potential factors that community stakeholders should consider for mitigating detrimental effects to neighborhood residents. Some of the policy options available to help ease pressures on renters in gentrifying neighborhoods are that state and local governments can provide affordable housing provisions in the form of rent subsidies or rent control, as well as tax abatement policies that allow landlords to close gaps between rising market rents and what they receive from long-term tenants [62]. Further, given our results suggesting residents generally want to remain in their neighborhoods, regardless of gentrification, developers and landlords can play a part in ensuring this is a possibility by providing tenant’s the first right to return to housing after any renovations, and state and regional policy makers can provide home purchasing assistance for those who wish to remain in the neighborhood as homeowners [62]. Additionally, considering some of the health impacts to residents from gentrified census tracts were thought to be due to resources leaving the neighborhood, local businesses, local policymakers and community-based organizations should consider ways for residents to continue accessing their services by either retaining presence in neighborhoods or providing other options, such as shuttles or travel vouchers for residents to continue accessing services if they move their organization to another neighborhood. Also, for new resources coming into a neighborhood, such as businesses looking to establish a presence in the neighborhood, consideration should be given to the range of residents represented to ensure new resources cater to the full range of the neighborhood population rather than just a sub-section of it. Finally, given our findings suggesting a mix of improvements and negative changes stemming from gentrification, some successful strategies that have been identified for ensuring more positive changes include community participation and bottom-up planning processes [13, 62–64]. To that effect, local elected officials and decision-making bodies should take greater efforts to involve community members in development decisions. In terms of extending this body of work, future research should explore whether similar outcomes can be seen in other geographical areas with different socio-demographic profiles. Further, studies should continue to explore and further refine terminology related to gentrification, biomarkers to track the impact of stress and other aspects of health in the context of gentrification and should continue to build out theory around the relationships between gentrification and health and wellbeing. Taken together, this study contributes important contextual information on previously observed differences in health and wellbeing for residents in gentrifying neighborhoods as well as insights that can help to inform policy in gentrifying spaces.

### Electronic supplementary material

Below is the link to the electronic supplementary material.

Supplementary Material 1

## Data Availability

Data sharing is not applicable to this article as no datasets were generated or analyzed during the current study.
