# Supplementary material for: Exploring differences in perceptions of gentrification, neighborhood satisfaction, social cohesion, and health among residents of two predominantly African American Pittsburgh neighborhoods (n = 60)
Source: BMC Public Health. 2023 Nov 1;23:2137. doi: 10.1186/s12889-023-16970-4 (PMC10621185; doi:10.1186/s12889-023-16970-4)
Supplement: Supplementary file 1 — Supplementary Material 1 [file 12889_2023_16970_MOESM1_ESM.docx]

**Supplementary Materials**

**Supplementary Table 1.** Exemplary quotes about gentrification

| **Domains** | **Participants from Gentrified Tracts** | **Participants from Non-Gentrified Tracts** |
| --- | --- | --- |
| Gentrification is occurring in the neighborhood | *High costs of living and residential turnover:* "You know, the homes is so expensive, a lot of times people of color can't afford it...the Hill used to be a mixture. It's a mixture now. But like I said, the homes, the people that bought these house, at first, they're not even there anymore. So, this is just different." (Stable resident, renter) | *Construction of new housing:* "There's a lot of that going on updating and revitalization [of housing]…It's taken a long time. I don't know if it's because of the government and the allocation of monies that they're allowing for certain time periods because that's what seems to happen." (Stable resident, homeowner) |
| Perceptions of gentrification | *Positive perception:* "I think that that's a good thing because, in order to interest people to come into the area, it has to look decent. And nobody wants to come in and live in beat-down stuff. So, I think that that's really good. I think that for your neighborhood to look nice you have to keep improving it." (Stable resident, homeowner) | *Not opposed to the idea of gentrification:* "Affordability is always my number one concern, but if I can benefit from the redevelopment or the reevaluation that they do in the community, I would be, I wouldn't be opposed to that either." (Mover, renter) |
|  | *Negative perception:* "I've been in the Hill all my life. So, stop chasing people who've been here all their lives out. You know, you build a new house and the person who's been living in the Hill can’t afford it anymore. Where are they supposed to go? They are pushing you out in the suburbs. That's not where I'm from. And that's not where I want to be." (Stable resident, renter) | *Good for some and bad for others: "*It's good and it's bad. It is good for some people. It’s bad for others. That's the thing, that's the rough part. Some people, like I said, the professional people, they can they can handle it. They can stay. They make the money and if you don't make that kind of money, you got to go." (Mover, renter) |
|  | *Wants to remain in neighborhood:* "I don't want to go to a different neighborhood, but I can't afford the rent because the rent right now is high." (Stable resident, renter) |  |
|  | *Wants to remain in neighborhood:* "I have no intentions of moving anywhere else. I was, like I said, pretty much born and raised in the Hill District and right now my plans are to stay, and I welcome the improvements." (Stable resident, homeowner) |  |
|  | *Wants to stay in neighborhood:* "We have to stay where we are because we already know how important the Hill District is. A lot of people, excuse me for saying this but a lot of light-skinned people want us dark-skinned people to move to East Liberty and different stuff like that. Well, we like to stay right where we are...Leave us where we're at. If you're going to come here, come to help, come to add on not to take away from." (Mover, renter) |  |

**Supplementary Table 2.** Exemplary quotes about neighborhood satisfaction

| **Domains** | **Participants from Gentrified Tracts** | **Participants from Non-Gentrified Tracts** |
| --- | --- | --- |
| Neighborhood trajectories | *Headed in a positive trajectory:* "Because just as you said, they’ve been building homes and everything looks nice. You'd be glad you live here. Because it looks so nice." (Stable resident, homeowner) | *Headed in a negative trajectory:* "I would say no because I don't see no redevelopment as far as like housing goes, like I said, the only store is Family Dollar, I would say this is maybe one of the communities that they haven't touched yet." (Mover, renter) |
|  | *Not good for everyone:* "They don't treat low income the way they treat the higher pay, rich people. And it's not supposed to be that. That's discrimination. And it happens every day, but we as black people are afraid of authority and will not speak up." (Mover, renter) | *Not good for everyone:* "Not for black people it's not in the right direction. There's nothing...they tear down, but they are not putting things back up but expensive housing that's not affordable for low income." (Mover, renter) |
| Most favorite neighborhood aspects | *Convenience:* "It’s convenient. I’m close to my mom and dad so that works out a lot and everything...I can walk practically anywhere I need to go. I can go to Oakland. I can go not too far to East Liberty but downtown and Oakland it’s great. I can actually walk there." (Stable resident, renter) | *Convenience:* "The convenience, everything that's around here. Don't have to leave, or go too far to get to what you need to get to." (Mover, renter) |
|  | *Housing improvements:* "They did the greatest thing. They remodeled. They turned apartments into houses. That was good. So, we can keep our grandkids and things. We got houses now up there. I felt really good about it, excellent. That was long time overdue. We all need it." (Mover, renter) |  |
|  | *Housing caveats:* "They didn't want to bring nobody up here on Section 8 for real, but they had to bring some people back and you can tell the difference where the people on the Section 8 side live and the people who pay that nice rent on the other side...this side is over here is all, whatever and then you look on the other side, like it's so quiet, it's all nice and clean and the grass is all nice...they don't really want too many black folks up here unless you really got some money." (Mover, renter) |  |
|  | *Housing caveats:* "Since they did the redevelopment and it’s no longer a low-income community, there’s no food banks local that comes to us because it’s mixed income." (Mover, renter) |  |
|  |  | *Sense of community:* "I love Homewood. I’m a native from Homewood. I just love the community period." (Mover, renter) |
| Least favorite neighborhood aspects | *Issues with violence:* "There's just a lot of violence up there, gun, fighting and physical." (Mover, homeowner) | *Issues with violence:* "The other end of Frankston there has been a whole lot of violence, shootings and all of that and fights and all that." (Stable resident, homeowner) |
|  | *Departure of the grocery store:* "I used to go right around the corner to the Shop and Save, and it's not there anymore. I can get in my car and go, but like I said, my oxygen don't last that long, so I'm always rushing to get back." (Stable resident, renter) | *Departure of the grocery store:* "I don't know why they took it away, the Shop n Save...I could walk instead of catching the bus…now I have to go further." (Stable resident, renter) |
|  | *Lack of resources:* "You ain’t got nowhere to eat over here and I think that’s terrible. That's my thing, stuff like that. Not even for these kids, they had no recreation like we had, they ain’t got nothing over here for these kids nothing and then you all want to sit up and talk about it. They hanging out, well they ain’t got nothing to do...When we were all kids, we had three recreation centers on one street on Bedford Avenue." (Stable resident, renter) | *Lack of resources:* "There was a lot for the kids to do, when I was growing up. And there's nothing for the kids to do now. They closed down the skating ring and the bowling alley. There's like no community centers around, there's nothing for the kids to do. So, all they do is, you know, standing on the corner, selling drugs, or smoking weed, or whatever it is they do. I just know they're not doing what I was doing. Others play video games. That's all they do now." (Mover, renter) |
|  | *Lack of resources:* "Let's say development we had was about approximately $50 million in development, residential. But when it comes to recreation, I think they got what, a 10 by 10 room to represent and a swimming pool that fit what, a dozen people...[It's] not adequate. When you think of a successful neighborhood, you know what I mean? When you think about how many units are in this area. And then you got three, two or three treadmills, or, you know what I mean?" (Stable resident, homeowner) | *Lack of resources:* "There's nowhere to walk to or any type of amenities...there are no stores at all. There's no close coffee shops. There's nowhere I can go and sit down and take my girlfriends... Most of my friends are white. So, there's nowhere in this area, that I can take my friends where they can feel comfortable, about being on this side of town being here." (Stable resident, homeowner) |

**Supplementary Table 3.** Exemplary quotes about social cohesion

| **Domains** | **Participants from Gentrified Tracts** | **Participants from Non-Gentrified Tracts** |
| --- | --- | --- |
| Interactions with neighbors | *Fewer neighborhood activities:* "I think that there's new housing, but there's not a lot of activities or things to do in a neighborhood. So, I think that people moving into the neighborhood are still doing things, wherever they came from because there's nothing to do here. So, you're really not meeting them and socializing with them...It was different before." (Stable resident, homeowner) |  |
|  | *Residential turnover:* "There's people moving out left and right up here. It's not the same community." (Mover, renter) | *Residential turnover:* "The people that moved in, moved down the streets, I have no idea who they are. I don't know names on them. I don't know anything." (Stable resident, homeowner) |
|  |  | *Lack of community togetherness:* "It's about the whole community coming together and stick together to make a change, we make a change when you come together, that's when things change, but when you separate, then you can’t resolve anything. So that's the main problem, people don't want to come together, they all compliant, but they don't want to come together." (Stable resident, renter) |
|  | *Newer neighbors are less friendly:* "They have the younger adults now moving into the neighborhood. So they’re different...a lot of them, aren’t friendly. I guess that's just the younger people’s way cuz I noticed a lot of the young people in their 20’s or whatever. I won’t say that they’re disrespectful, but, you know, they’re – I'm used to, when you see somebody on the street, you say, hello. Then keep moving. Well, the neighborhood around doesn’t do that, you know? " (Stable resident, homeowner) |  |
|  | *Less friendly neighbors:* "It used to be, people in the neighborhood looked out for each other, you know? I mean, but I guess everybody got so standoffish. I was telling you like my nephew passed away...he was just getting out of his car and just passed out. On the street, nobody came around...They stood across the street where I live, just looking...But they didn’t say anything." (Stable resident, homeowner) |  |
|  | *Torn down homes:* "The main thing of the street where I live one, it's not really a neighborhood here anymore because most of the houses are gone." (Stable resident, homeowner) |  |
|  | *Stricter building rules:* "The new housing, so you get into these housing that they built, they limited what your kids can do completely. They limited, like if they got a parking lot or something around their housing units, the kids can’t ride their bikes anymore...It’s like you’re imprisoned inside your own little cute apartment." (Mover, renter) |  |
| Community Engagement | *Likes to engage:* "I think my biggest thing is my church, I am part of a church and our church does a lot of things in the neighborhood. You know, we try to have little programs for children. You know, do homework help. You know, we do a lot of things for children and seniors." (Stable resident, homeowner) | *Likes to engage:* "I was part of one of their group things. That’s what’s about the housing and the planting...We was in that program where they say what they’re going to do with Homewood – that kind of stuff." (Stable resident, homeowner) |
|  | *Does not like to engage:* "I don’t go to other things because there there’s a bunch of bull crap. They don't do that. But it’s bull crap. I don't believe nothing they say. Nothing they say, because it's a bunch of bull. Oh, everybody is trying to get what they want. I don't believe in that. They are not for the neighborhood; they are for they self." (Stable resident, homeowner) | *Does not like to engage:* "Hmm I don't know. I'm not sure. I haven't really done that get involved in the community. That's something new to me, I just basically I leave people alone and just say hi and goodbye. That's all I say go about my business" (Mover, renter) |
|  | *Difficult to engage:* "I think that there are changes happening. But I think that the meetings are held at strange times, so everybody can't participate in them. And I think that it's a handful of people who are making decisions or suggesting things, but it's not open to the community. It's very hard to find out about things. And, when you do find out, everything's already in gear." (Stable resident, homeowner) | *Would like more opportunities to get involved:* "I mean, if there were things to do, I would participate. I feel like that's how you get to know your neighbors, your community. That's like how you give back to your community. So, I would definitely participate." (Mover, renter) |
|  | *No voice in the community:* "I guess I'm so used to not having a voice in this neighborhood. You know, it doesn't bother me." (Stable resident, homeowner) | *Unsure of whether they have a voice:* "No, I never tried, all I do is go up the street and vote and come on home. I don't try to use my voice in the neighborhood." (Mover, renter) |
|  | *Decisionmakers do not consider residents' voices:* "I feel as though I can voice and whatever like that, we can all voice, but come down to the end, they going to do what they want to do. They're going to do what they want." (Mover, renter) | *Decisionmakers do not consider residents' voices:* "I don't feel like they listen to the actual people that are in a community and I'm just certainly going to speak up for that as far as the neighbors that I knew that do go to the block meetings or something about the community, basically, our voices is falling on deaf ears." (Stable resident, homeowner) |

**Supplementary Table 4.** Exemplary quotes about health changes

| **Domains** | **Participants from Gentrified Tracts** | **Participants from Non-Gentrified Tracts** |
| --- | --- | --- |
| Reasons for health changes | *Health changes due to moving for renovations:* "Living in the projects can make you or break you...And it plays a little on your psyche...when I found out I was able to move back up to Skyline [after renovations], I was never so happy. It seemed like the whole time that I lived up in Chauncey, there was a shooting every day, every day, there was some type of violence. I remember on Christmas Eve; they had a whole shootout in my court...they shot up the steps and the concrete steps fell. Like it was so much crazy. A guy I went to school with, I watched him die outside...And I saved someone from dying. Like it was a lot." (Mover, renter) | *Health changes due to moving:* "There's less stress now. I can get a lot more around here than I could in Homewood." (Mover, renter) |
|  | *Health changes due to neighborhood resources:* "Everything stresses me...I got a touch of depression and anxiety...There’s really no places, nothing to do in Hill District for me." (Mover, renter) |  |
|  |  | *Health changes due to violence:* "I be depressed and stuff...sometime I be like no, like watch your back when you get your groceries, I'm not being funny. I mean you just got to be careful period because no gun, no bullet don’t have none of that they don't care who they ask or whatever." (Stable resident, renter) |
|  |  | *Health changes due to safety issues:* "I used to walk a lot more and I don't...there was a thing on my app, on my phone where they have had a lot of robbery and that there's been a lot of robberies. And I didn't realize that. So, I'm just trying to be more cautious now about the area." (Stable resident, homeowner) |
